# Serum markers of the extracellular matrix remodeling reflect antifibrotic therapy in bile-duct ligated rats

**DOI:** 10.3389/fphys.2013.00195

**Published:** 2013-07-30

**Authors:** Robert Schierwagen, Diana J. Leeming, Sabine Klein, Michaela Granzow, Mette J. Nielsen, Tilman Sauerbruch, Aleksander Krag, Morten A. Karsdal, Jonel Trebicka

**Affiliations:** ^1^Department of Internal Medicine I, University of BonnBonn, Germany; ^2^Fibrosis Biology and Biomarkers, Nordic BioscienceHerlev, Denmark; ^3^Department of Gastroenterology, Odense University Hospital, University of Southern DenmarkOdense, Denmark

**Keywords:** fibrosis, statins, markers, antifibrotic, ECM (extracellular matrix), remodeling

## Abstract

**Background:** Progression of liver fibrosis is characterized by synthesis and degradation of extracellular matrix (ECM). Matrix-metalloproteinases (MMP) cleave collagen fibers at a specific site and thereby generate soluble fragments of ECM (neo-epitopes). The levels of these neo-epitopes might reflect the stage of liver fibrosis and may allow monitoring of anti-fibrotic therapies. Here we analyzed these neo-epitopes as read-out for a liver directed therapy with statins.

**Methods:** Bile duct ligation (BDL) was performed on wild type rats, which received atorvastatin (15 mg/kg^*^d) for 1 week starting at 1, 2, 3, 4 and 5 weeks after BDL (T1–T5), while controls remained untreated. Hepatic fibrosis was analyzed by immunohistochemistry and hepatic hydroxyproline content. TGFβ levels were measured by RT-PCR. Proteolytic activity of MMP-2 was examined by zymography. Levels of degradation MMP driven type I, III, IV and VI collagen degradation (C1M, C3M, C4M, and C6M) and type III and IV collagen formation (PRO-C3 and P4NP7S) markers were assessed by specific ELISAs in serum probes.

**Results:** Serum markers of ECM neo-epitopes reflected significantly the deposition of ECM in the liver and were able to distinguish between early (T1–T3) and severe fibrosis (T4–T5). Statin treatment resulted in reduction of neo-epitope markers, especially when therapy was started in the stage of severe fibrosis (T4–T5). Furthermore, these markers correlated with hepatic expression of profibrotic cytokines TGFβ1 and TGFβ2. Formation markers of type III and IV collagen (PRO-C3 and P4NP7S) and degradation markers C4M and C6M correlated significantly with hepatic MMP-2 activity in rats with severe fibrosis.

**Conclusion:** Determination of ECM remodeling turnover markers in serum allowed a distinction between mild and severe fibrosis. With respect to statin therapy, the markers may serve as read-out for efficacy of anti-fibrotic treatment.

## Introduction

Progressive liver fibrosis is a consequence of chronic hepatic inflammation from various causes (Friedman, 2003). The ultimate result is cirrhosis and end-stage liver disease, which has a huge impact on the patient's morbidity and mortality as well as medical and financial resources world-wide (Brundtland, 2002). The key-process during progression of liver fibrosis is the synthesis and deposition of extracellular matrix (ECM), which mainly consists of different collagen types. The responsible cells for scar-formation in the liver are hepatic myofibroblasts, mainly deriving from hepatic stellate cells. These are activated and stimulated by the ongoing inflammation in the liver which is associated with formation of profibrotic cytokines including transforming growth factor-β (TGFβ) (Friedman, 2001, 2008; Pinzani, 2002; Bataller and Brenner, 2005). However, it is important to emphasize that fibrosis progression is dynamic and constitutes both formation and degradation of ECM, which becomes imbalanced during liver disease (Gressner and Weiskirchen, 2006; Friedman, 2008; Parola et al., 2008). During this remodeling process endopeptidases such as matrix-metalloproteinase-2 and -9 (MMP-2 and -9) are upregulated and are able to degrade excessive ECM (Barascuk et al., 2010). Small ECM protein fragments are released and may enter the circulation and may serve as systemic biochemical markers of hepatic ECM remodeling. The sites of degradation by specific MMPs are distinct and are known as neo-epitopes. These neo-epitopes can be measured using novel protein finger-print techniques (Karsdal et al., 2010, 2012).

Only few clinical trials have been conducted to test new anti-fibrotic drugs in patients with liver diseases, partly because reliable parameters for long-term monitoring of the therapeutic effect are lacking. Liver biopsy, as a widely accepted technique, is invasive and shows problems of gaining representative samples as well as the inter-observer variation (Bedossa et al., 2003). Transient elastography is non-invasive and a more and more accepted tool to assess fibrosis. Nevertheless, this technique carries restrictions related to cholestasis, obesity, severe inflammation and dialysis (Coco et al., 2007; Arena et al., 2008; Millonig et al., 2008, 2010; Koch et al., 2011; Trabut et al., 2012). Therefore, additional markers reflecting hepatic fibrotic activity and therapeutic effects of antifibrotic agents are needed.

Thus, the present study aims to validate novel serological markers of ECM remodeling (ECMR) that reflect hepatic fibrotic activity at different stages of fibrosis after bile-duct ligation in rats (I), and to investigate whether they mirror the therapeutic effect of HMG-CoA-reductase inhibitors (II) (Trebicka et al., 2010; Klein et al., 2012).

## Materials and methods

### Animals and treatment regimens

**Figure 1:**
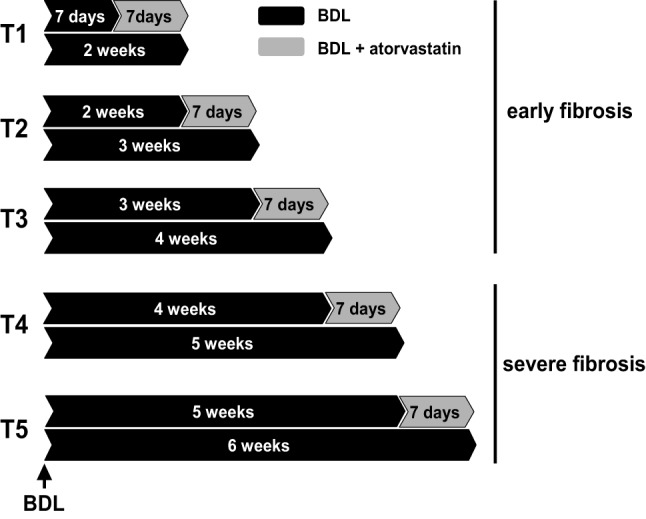
**Experimental design**. Bile duct ligated (BDL) rats were used with or without atorvastatin treatment for 1 week at different times after BDL. T1 to T3 were pooled in group early fibrosis. T4–T5 were pooled in group severe fibrosis. Each group with a minimum of *n* = 7.

Sprague-Dawley rats with an initial body weight of 180–200 g were used. Twenty-four rats underwent BDL as previously described (Trebicka et al., 2007, 2010; Klein et al., 2012). Twenty-one rats underwent BDL and were additionally treated with a specific dosage of atorvastatin (Trebicka et al., 2007, 2010; Klein et al., 2012) (15 mg/kg body weight per day). This study was authorized by the local committee for animal studies (Bezirksregierung Köln, 50.203.2-BN22, 46/05). All rats received chow and water *ad libidum*. Atorvastatin was administrated as described (Trebicka et al., 2007, 2010; Klein et al., 2012). The rats were daily weighed and pair-fed. The treatment regimens are shown in Figure 1. Respective BDL rats received atorvastatin chow for 1 week starting on distinct time points after BDL, 1 (T1), 2 (T2), 3 (T3), 4 (T4) or 5 (T5) weeks after BDL. BDL control rats were sacrificed at the corresponding time points after BDL.

### Tissue collection and biochemical analysis

Animals were sacrificed after the described time points, blood was withdrawn to measure biochemical parameters (ALT, AP, AST, bilirubin, creatinine and γ GT) and plasma samples were isolated using standard methods. Liver samples were cut into fragments and either snap-frozen in liquid nitrogen and stored at −80°C or preserved in formaldehyde as described previously (Trebicka et al., 2007, 2008, 2010).

### Hepatic hydroxyproline content

Hepatic hydroxyproline was determined photometrically in liver hydrolysates (Trebicka et al., 2007, 2010). Segments (200 mg) of snap-frozen livers were hydrolyzed in HCl (6 N) at 110°C for 16 h and filtered. Aliquots (50 μl) from each sample were incubated with chloramine T (2.5 mM) for 5 min and Ehrlich's reagent (410 mM) for 30 min at 60°C. Absorption was measured three times at 558 nm. For determination of hydroxyproline concentration a standard curve for hydroxyproline was used. Results are expressed as μg/g of wet liver tissue.

### Sirius-red staining, αSMA-immunohistochemistry and morphometry

Liver specimens were fixed in 10% formalin. As described previously (Trebicka et al., 2007, 2010), paraffin-embedded sections (2–3 μm) were stained in 0.1% Sirius-red F3B in saturated picric acid (Chroma, Münster, Germany) to detect collagen fibers.

For immunohistochemical staining of α-smooth muscle actin (αSMA) sections were incubated with an mouse-anti-αSMA antibody (clone 1A4, Sigma-Aldrich, St. Louis, USA) diluted 1:600 in Tris-buffered saline for 60 min. A biotinylated rabbit-anti-mouse antibody, absorbed wit rat serum (Dako, Glostrup, Denmark), was used as secondary antibody and applied subsequently diluted 1:200 for 45 min. Streptravidin-conjugated alkaline phosphatase (1:200, 45 min; Dako) was then attached to the complex. Sections were finally developed with new fuchsin-naphtol AS-BI (Sigma-Aldrich) and counterstained with hematoxyline.

Stereomicroscopic analysis was performed as described previously (Trebicka et al., 2007, 2010). On sections with a surface of 1 cm^2^ five hundred points were counted on a magnification of 400x. On Sirius-red staining test points were classified as connective tissue (positive staining), as well as hepatocytes, bile ducts or other structures (not stained). αSMA positive cells were counted on αSMA-immunohistological sections and characterized by their location as sinusoidal, periductular, portal/septal and others (e.g., vascular smooth muscle cells). The volume fraction of each structure was calculated as percentage of points overlaying the structure in relation to the total number of counted points.

### Quantitative RT-PCR

RNA was isolated from liver segments of 30 mg using the RNeasy kit (Qiagen, Basel, Switzerland) with DNase treatment (Promega, Wallisellen, Switzerland) followed by reverse transcription with M-MLV reverse transcriptase (Invitrogen, Basel, Switzerland) according to the manufacturers' instructions. Quantitative PCR was carried out on the ABI 7700 sequence detector (Applied Biosystems, Rotkreuz, Switzerland) with previously described sequences and accession numbers (Trebicka et al., 2010). Gene expression values were calculated based on ΔC_*t*_ method (Trebicka et al., 2007, 2010; Klein et al., 2012) and normalized to expression of GAPDH. Results were calculated as 2^−ΔΔ*Ct*^ and express the x-fold increase of gene expression compared to sham operated rats.

### MMP-2 zymography

Proteolytic activity of tissue homogenates was examined by gelatine zymography as previously published (Trebicka et al., 2010). The supernatants of liver homogenates (20 μg of protein) were subjected to 10% SDS-PAGE using gels containing 0.3% gelatine. Proteolytic bands of 62 and 65 kDa, corresponding to the active and latent form of MMP-2 respectively were quantified by densitometry using AIDA software (Raytest, Urdorf, Switzerland).

### Enzyme linked immunosorbent assay (ELISA)

Serological markers for MMP degradation of ECM proteins C1M (type I collagen), C3M (type III collagen), C4M (type IV collagen), C6M (type VI collagen), and formation markers PRO-C3 (type III collagen), P4NP 7S (type IV collagen) were assessed according to protocol in plasma of each rat using technical robust enzyme linked immunosorbent assays as described previously (see Table 1) (Barascuk et al., 2010; Veidal et al., 2011a,b; Leeming et al., 2011, 2012; Nielsen et al., 2013). Each marker was stratified according to time of BDL and atorvastatin treatment. The serum was obtained from previously analyzed animals (Trebicka et al., 2010).

**Table 1:** **Overview of used degradation and formation markers of ECM**.

**Assay name**	**Target**	**Antibody type**	**Detection range (ng/mL)**	**Intra-assay variation (%)**	**Inter-assay variation (%)**	**References**
C1M	MMP-2/9/13 degraded type I collagen	Monoclonal	0.83–500	10.1	6.7	Leeming et al., 2011
C3M	MMP-9 degraded type III collagen	Monoclonal	0.9–50	4.7	6.5	Barascuk et al., 2010
C4M	MMP-2/9 degraded type IV collagen	Monoclonal	0.6–100	4.8	12.1	Veidal et al., 2011a
C6M	MMP-2/9 degraded type VI collagen	Monoclonal	0.3–250	4.1	10.1	Veidal et al., 2011b
PRO-C3	N-terminal propeptide of type III collagen	Monoclonal	0.9–200	4.1	11.0	Nielsen et al., 2013
P4NP 7S	7S domain of type IV collagen	Monoclonal	7.9–500	9.7	11.7	Leeming et al., 2012

### Statistical analysis

Data are presented as mean ± standard error of the mean (SEM). Mann–Whitney-*U*-test or Wilcoxon was used for comparison between groups as appropriate. For computation of the correlations SPSS (Chicago, USA) was used. Two sided *p*-values <0.05 were considered statistically significant.

## Results

### Serum levels of ECM remodeling markers mirrored the quantity of hepatic fibrosis in BDL rats

The serum levels of all ECMR markers of degradation and formation of ECM-proteins were negatively correlated with the hepatocyte mass (Table 2). This correlation was observed in all rats, as well as in severe fibrosis regardless of treatment (Table 2). Of note there was no correlation of any ECMR marker with creatinine and therefore these markers were not influenced by renal function (data not shown).

**Table 2:** **Correlations of hepatic histology and function with ECM markers**.

		**Biomarker**					
		**C1M**	**C3M**	**C4M**	**C6M**	**PRO-C3**	**P4NP 7S**
		***r***	***r***	***r***	***r***	***r***	***r***
Hepatocytes (staining)	All animals	−0.393^**^	−0.318^*^	−0.346^*^	−0.549^**^	−0.424^**^	−0.529^**^
	Untreated animals	−0.563^**^	−0.391^*p* = 0.065^	−0.538^**^	−0.553^**^	−0.373^*p* = 0.079^	−0.479^*^
	Atorvastatin treated animals	−0.548^*^	−0.618^**^	−0.551^*^	−0.670^**^	−0.565^*^	−0.682^**^
ECM (staining)	All animals	0.386^*^	n.s.	0.355^*^	0.586^**^	0.511^**^	0.566^**^
	Untreated animals	0.577^**^	0.451^*^	0.583^**^	0.664^**^	0.590^**^	0.628^**^
	Atorvastatin treated animals	0.424^*p* = 0.090^	0.518^*^	n.s.	0.560^*^	0.540^*^	0.550^*^
Hepatic hydroxyproline content	All animals	n.s.	n.s.	n.s.	0.445^**^	0.403^*^	0.464^**^
	Untreated animals	0.699^**^	0.626^**^	0.779^**^	0.818^**^	0.683^**^	0.681^**^
	Atorvastatin treated animals	0.631^*p* = 0.050^	n.s.	0.716^*^	0.662^*^	0.596^*p* = 0.069^	0.532^*p* = 0.114^

Significance of correlations were defined by non-parametric testing (n=minimum 7/group) and p<0.05 was considered significant. Data are presented as Pearson correlation coefficient (r) of ECM or hepatic hydroxyproline content with ECMR markers (C1M, C3M, C4M, C6M, PRO-C3 and P4NP7S). Asterisks indicate statistical significant correlations. (

* p < 0.05,

** p < 0.01, n.s, not significant).

**Figure 2:**
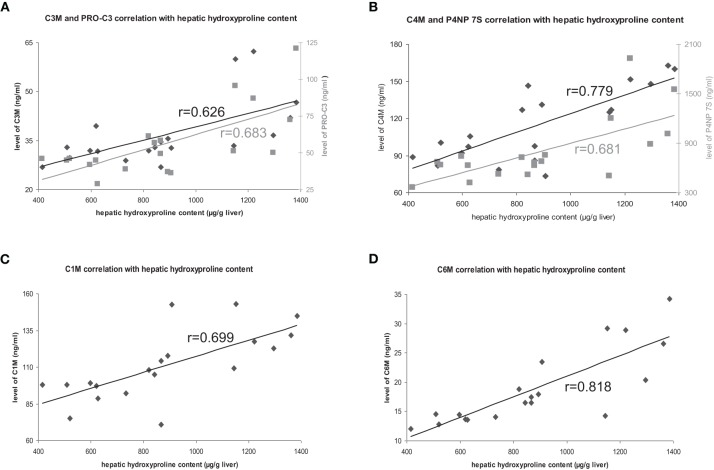
**Correlation of hepatic hydroxyproline content with ECM markers**. Correlations with degradation markers shown in black, correlations with formation markers shown in gray.

The deposition of total collagen, quantified by Sirius-red staining and hydroxyproline content in untreated rats correlated with all markers highly significant with ECM amount (Table 2, Figure 2).

Furthermore, the levels of the ECMR markers in all animals with different fibrosis severity (T1–T5), as well as in severe fibrosis (T4, T5) correlated with the hepatic collagen amount regardless of atorvastatin treatment (Table 2). However, these correlations were not as strong as in untreated animals, suggesting that atorvastatin treatment had an effect on the levels of these neo-epitope markers differentially to the hepatic collagen quantity determined by staining and hydroxyproline content.

The levels of collagen formation assessed serologically (PRO-C3 and P4NP7S), as well as of the collagen degradation markers C3M and C6M were significantly lower in early fibrosis (T1–T3) compared to late stages of fibrosis (T4, T5) independent of statin treatment as shown in Table 3.

**Table 3:** **Levels of ECM markers in blood**.

**Group**	**C1M (ng/ml)**	**C3M (ng/ml)**	**C4M (ng/ml)**	**C6M (ng/ml)**	**PRO-C3 (ng/ml)**	**P4NP7S (ng/ml)**
T1	95.01 ± 5.16	34.02± 2.33	100.17 ± 4.37	12.32 ± 0.60	36.51 ± 3.50	535.64 ± 55.29
T2	113.95 ± 8.72	37.73 ± 2.63	115.84 ±9.77	18.12 ± 1.37^b^	50.86 ± 2.85^b^	669.76 ± 32.23
T3	114.40 ± 7.64	40.15 ± 5.26	119.41 ± 8.79	15.30 ± 1.20	43.01 ± 4.86	592.13 ± 78.00
early fibrosis	108.01 ± 4.62	37.32 ± 2.12	111.95 ± 4.96	15.35 ± 0.79	43.73 ± 2.45	601.69 ± 34.46
T4	125.25 ± 16.76	46.97 ± 8.18	127.50 ± 17.53	21.29 ± 3.21^a^	56.03 ± 9.85	932.55 ± 131.06^a^
T5	117.12 ± 8.73	46.09 ± 4.56^a^	118.77 ± 10.53	21.52 ± 2.42^b^	62.29 ± 9.15^a^	989.46 ± 140.45^a^
Severe fibrosis	120.47 ± 8.66	46.45 ± 4.31^c^	122.37 ± 9.57	21.43 ± 1.94^d^	59.72 ± 6.78^c^	966.02 ± 98.92^d^

Each point in time consist of BDL rats with and without atorvastatin treatment. Points in time T1–T3 are pooled in group early fibrosis. Points in time T4–T5 are pooled in group severe fibrosis. Data are means ± standard errors for all points of time. Letters a to d indicate statistical significance. (

a , p < 0.05 vs. T1;

b , p < 0.01 vs. T1;

c , p < 0.05 vs. early fibrosis,

d , p < 0.01 vs. early fibrosis).

Summarizing these data, the markers reflect ECM deposition in the liver and discriminate between early and severe fibrosis.

### Serum levels of ECM remodeling markers analysed on weekly basis after BDL

**Figure 3:**
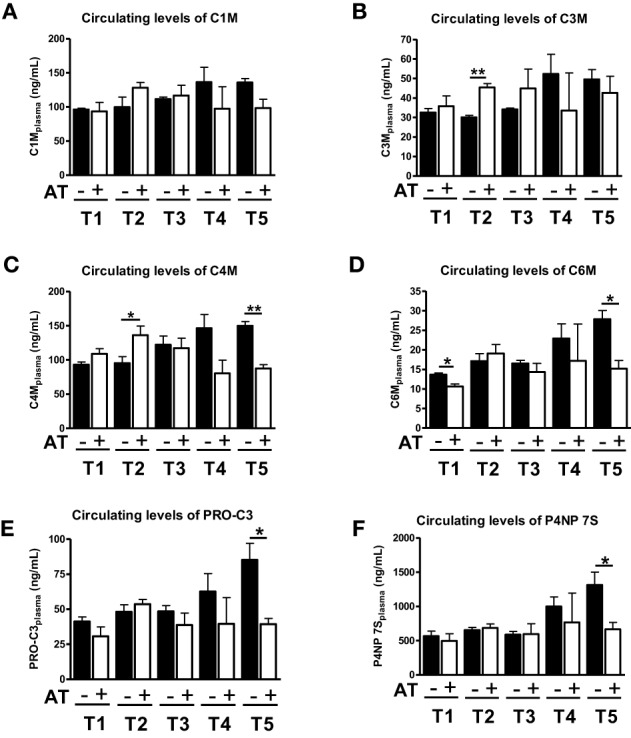
**Levels of ECM markers in blood with (+AT) or without (–AT) atorvastatin treatment**. BDL rat liver fibrosis model with atorvastatin intervention 1 (T1), 2 (T2), 3 (T3) and 5 (T5) weeks post BDL surgery. Plasma levels of C1M, C3M, C4M, C6M, PRO-C3, and P4NP 7S levels are shown as mean levels ± standard errors for all time points. Asterisks indicate statistical significance as indicated by bars. (^*^p < 0.05, ^**^p < 0.01).

We have previously shown that 1 week of atorvastatin treatment prevented ECM deposition in the early stages of fibrosis (T1–T3), but did not change the amount of ECM significantly in severe fibrosis (Trebicka et al., 2010). Interestingly, the ECRM markers provided a slightly different picture. When statin treatment was started 1 week after BDL (T2) the degradation markers for type III and IV collagen increased in serum of these animals (Figures 3B,C), whereas in the early fibrosis (T1) the levels of the degradation marker for collagen type VI (C6M) decreased significantly (Figure 3D). The levels of degradation marker type I collagen and formation markers for type III and IV were not significantly influenced in early fibrosis (Figures 3A,E,F).

In severe fibrosis (T4, T5), the levels of all neo-epitopes for degradation and formation show the same trend, since they are decreased by atorvastatin treatment (Figure 3). Especially in T5, we observed a statistical significant decrease in serum levels of C4M, C6M, PRO-C3, P4NP7S (Figures 3C–F). In the T5 group we have previously observed an effect of atorvastatin on the turnover of activated HSC and secretory activity of myofibroblasts due to induction of senescence in these cells (Trebicka et al., 2010; Klein et al., 2012).

### Levels of ECM remodeling markers in serum of BDL rats treated for 1 week with atorvastatin in early and severe fibrosis

The effects of atorvastatin could be divided into basically two different types: on the one side the early effect on the prevention of activation of HSC (early stages of fibrosis) and on other side the blunting of activity and induction of senescence in myofibroblasts (severe stages of fibrosis). We, therefore, cumulatively analyzed the levels of these marker in early and severe fibrosis (Trebicka et al., 2010; Klein et al., 2012).

**Figure 4:**
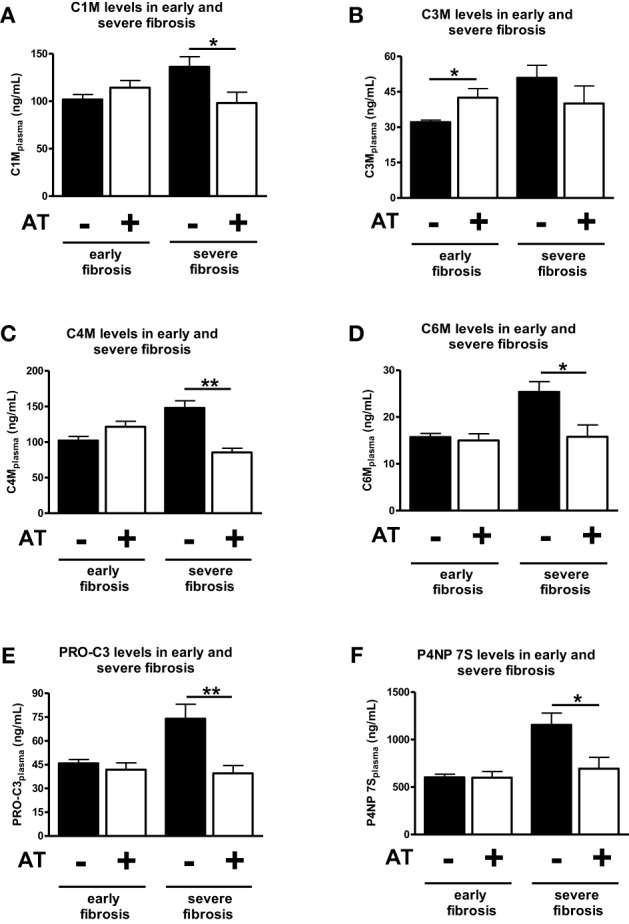
**Comparison of early and severe fibrosis for all ECM markers in atorvastatin treated or untreated animals**. Different points of time were pooled into groups early fibrosis (T1–T3) and group servere fibrosis (T4 and T5) and separated by statin treated (+AT) and untreated (−AT) animals. Asterisks indicate statistical significance as indicated by bars. (^*^p < 0.05, ^**^p < 0.01).

Analyzed together atorvastatin treatment for 1 week did not changed significantly the levels of ECM degradation or formation in early fibrosis (1, 2, or 3 weeks after BDL) (Figures 4A,C–F). The only exception was the degradation neo-epitope for collagen type III, which increased after atorvastatin (Figure 4B). In a previous study these early stages showed significant lower hydroxyproline content and Sirius-red staining areas after atorvastatin treatment (Trebicka et al., 2010). By contrast, the serum levels of neo-epitopes for degradation and formation of ECM decreased after 1 week of atorvastatin treatment at late stages of fibrosis (after 4 and 5 weeks of BDL) (Figure 4). These data suggest that these novel ECM degradation and formation markers might reflect the amount of fibrosis, as well as the effect of an anti-fibrotic treatment before the amount of ECM changes notably in the liver.

### Serum levels of neo-epitopes of ECM correlated with hepatic expression of profibrotic cytokines

**Figure 5:**
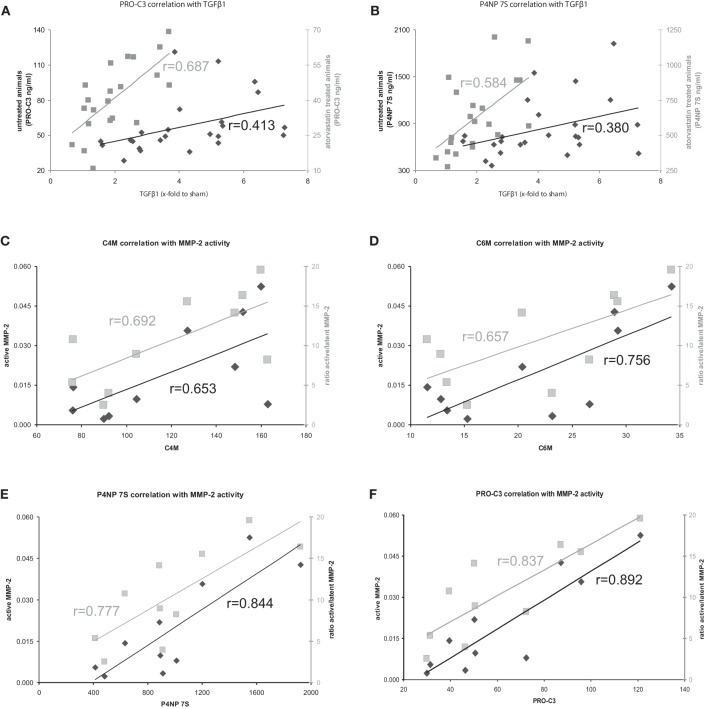
**(A,B)** Correlation of formation markers with profibrotic cytokine TGFβ1 in atorvastatin treated or untreated animals. Correlations with untreated animals shown in black, correlations with atorvastatin treated animals shown in gray. **(C–F)** Correlation of ECM turnover with ECM markers in severe fibrosis. Correlations with active MMP-2 shown in black, correlations of ratio active/latent shown in gray.

Hepatic mRNA levels of TGFβ1 and TGFβ2 in BDL rats were highly correlated with the serum levels of degradation markers for type I and IV collagen, as well as formation markers for type III and IV collagen, regardless of treatment (Table 4, Figures 5A,B). Interestingly, the hepatic mRNA levels of TGFβ1 and TGFβ2 showed strong correlation with almost all these neo-epitopes of ECM, when the early or the severe fibrosis group was observed separately. Similarly, strong correlations of TGFβ1 and TGFβ2 hepatic mRNA levels were observed with these markers when the treated and untreated animals were analyzed separately (Table 4; Figures 5A,B). Of note, atorvastatin treatment decreased the hepatic levels of these cytokines in severe fibrosis stages of BDL animals as shown previously (Trebicka et al., 2010), similarly to the now described levels of neo-epitopes (Figure 4).

**Table 4:** **Correlations of mRNA levels from profibrotic cytokines with ECM markers**.

		**Biomarker**				
		**C1M**	**C4M**	**C6M**	**PRO-C3**	**P4NP 7S**
		***r***	***r***	***r***	***r***	***r***
	All animals	0.382^**^	0.485^**^	0.506^**^	0.583^**^	0.505^**^
TGFβ1 mRNA (x-fold)	Untreated animals	0.359^*p* = 0.085^	0.541^**^	0.420^*^	0.413^*^	0.380^*p* = 0.067^
	Atorvastatin treated animals	0.536^*^	0.379^*p* = 0.090^	0.372^*p* = 0.097^	0.687^**^	0.687^**^
	Early fibrosis	n.s.	n.s.	0.384^*^	0.576^**^	n.s.
	Severe fibrosis	0.505^*^	0.723^**^	0.526^*^	0.560^*^	0.604^*^
TGFβ2 mRNA (x-fold)	All animals	0.345^*^	0.353^*^	0.496^**^	0.488^**^	0.512^**^
	Atorvastatin treated animals	0.515^*^	n.s.	0.454^*^	0.526^*^	0.646^**^
	Early fibrosis	0.304^*p* = 0.115^	n.s.	0.427^*^	0.425^*^	0.332^*p* = 0.085^
	Severe fibrosis	n.s.	0.556^*^	0.508^*^	0.508^*^	0.622

Significance of correlations were defined by non-parametric testing (n = minimum 7/group) and p < 0.05 was considered significant. Data are presented as Pearson correlation coefficient (r) of mRNA levels of profibrotic cytokines (TGFβ1 and TGFβ2) with respective ECMR markers (C1M, C4M, C6M, PRO-C3 and P4NP 7S). Asterisks indicate statistical significant correlations. (

* p < 0.05,

** p < 0.01, n.s, not significant).

### The serum levels of ECM remodeling markers reflect hepatic ECM turnover in BDL induced fibrosis

The serum levels of the degradation marker for type IV and VI collagen, as well as formation markers for type III and IV collagen correlated strongly and significantly with hepatic MMP-2 levels measured by zymography in the rats with severe fibrosis (Figures 5C–F). Active MMP-2 and its activity calculated as the ratio of active to latent MMP-2 correlated strongly with all degradation and formation markers in severe fibrosis, pointing out that ECM markers display the turnover of ECM in liver disease (Table 5, Figures 5C–F).

**Table 5:** **Correlation of ECM turnover with ECM markers**.

		**Biomarker**			
		**C4M**	**C6M**	**PRO-C3**	**P4NP 7S**
		***r***	***r***	***r***	***r***
MMP-2 lat	severe fibrosis	0.576^*p* = 0.081^	0.715^*^	0.840^**^	0.835^**^
MMP-2 akt	severe fibrosis	0.653^*^	0.756^*^	0.892^**^	0.844^**^
MMP-2 akt/lat	severe fibrosis	0.692^*^	0.657^*^	0.837^**^	0.777^**^

Significance of correlations were defined by non-parametric testing (n = minimum 7/group) and p < 0.05 was considered significant. Data are presented as Pearson correlation coefficient (r) of MMP-2 (latent, active and ratio of active to lantent) with respective ECM markers (C4M, C6M, PRO-C3 and P4NP7S). Asterisks indicate statistical significant correlations. (

* p < 0.05,

** p < 0.01, n.s, not significant).

## Discussion

The present study demonstrates that novel serum markers of ECMR reflect the dynamic process of fibrosis and may thereby used to assess effects of antifibrotic therapies.

Liver fibrosis is usually a slow progressing disease and it may take decades to develop first symptoms and complications. In the end-stage of liver disease, liver cirrhosis, the patients show many severe complications and may decompensate rapidly. At these stages the only therapeutic options are liver transplantation and/or treatment of the complications. Therefore, attenuation or even interruption of fibrosis progression is an important therapeutic goal. Therapy varies dependent on etiology of disease and during therapy—no matter which—it would be most desirable to monitor its effect on the fibrotic process.

The tissue ECMR was assessed using newly developed ELISAs, that utilize monoclonal antibodies raised against specific degradation fragments unique for ECM proteins that are involved in the development of liver fibrosis and are remodeled by disease relevant proteases. They may carry unique disease “fingerprints,” why this approach is coined Protein Fingerprint Technology (Karsdal et al., 2009, 2012; Barascuk et al., 2010; Vassiliadis et al., 2011; Veidal et al., 2011a,b; Leeming et al., 2012, 2013). These markers have been validated in different models and severities of fibrosis in previous studies and compared to control non-fibrotic rats (Karsdal et al., 2009, 2012; Barascuk et al., 2010; Vassiliadis et al., 2011; Veidal et al., 2011a,b; Leeming et al., 2012, 2013).

In our BDL model we observed higher levels of these markers in early fibrosis, compared to severe fibrosis (Table 3). Moreover the levels of ECMR markers correlated very strongly with the amount of ECM, especially in the untreated BDL group. In these untreated animals, the fibrosis progression was assessed on weekly basis after BDL, as previously described (Trebicka et al., 2010). This on the one hand confirms previous findings and on the other hand validates our present findings in this rat model (Table 2).

The mechanisms of progressing fibrosis have been widely investigated in animal models, and many strategies have been published to blunt or attenuate fibrosis. However, in humans only a few trials have tested anti-fibrotic agents. One important reason for this is that animals can be treated concomitantly with initiation of hepatic injury, which makes it easier to assess the effect in the amount of fibrosis accumulated. In early fibrosis, it is easy to assess differences in the amount of fibrosis, as shown in our animal model (Trebicka et al., 2010). In severe fibrosis, the total amount of fibrosis is inadequate for the assessment of the treatment success. Atorvastatin prevents the accumulation of ECM, when it is administered in early phases of fibrosis in a BDL model of rats (Trebicka et al., 2010). Indeed, atorvastatin inhibited the activation of hepatic stellate cells *in vitro* the main producers of ECM in early phases of fibrosis (Klein et al., 2012).

The clinical situation is different; the patients come for treatment when fibrosis is severe and clinically evident. In these severe fibrosis the total amount of ECM does not change rapidly, therefore a long-term follow-up are needed to assess treatment effects. In our model, we mimicked this situation. In severe fibrosis (4 and 5 weeks after BDL) the rats were treated for 1 week with atorvastatin. The amount of ECM did not change significantly, but we observed a significant effect on the remodeling of ECM, since MMP-2 activity was blunted, the expression of profibrotic cytokines TGFβ1 and TGFβ2 were decreased (Trebicka et al., 2010; Klein et al., 2012). The serum levels of ECMR markers correlated strongly with TGFβ levels, as well as with active MMP-2 and MMP-2-activity, assessed as the ratio of active to latent MMP-2 (Tables 4, 5, Figures 5C–F). This demonstrates that the levels of these markers mirror early changes in the fibrosis progression, even before the ECM amount is changed. This is the first evidence that serum levels of these ECMR markers as read-out for anti-fibrotic treatment. The comparison of the accuracy of these markers with other serological fibrosis markers and the investigation in other models of fibrosis should be topic of future studies.

In severe fibrosis statin treatment decreased the turnover of activated hepatic stellate cells (Trebicka et al., 2010), due to the induction of senescence in these profibrogenic cells (Klein et al., 2012). In the present study, we showed that these profibrogenic cells produce less collagen (formation markers) and the turnover of ECM is reduced as well (as shown by reduced degradation markers), which led to the observation that in the severe fibrosis statin treatment for 1 week did not decrease the static amount of total collagen in the liver assessed by hydroxyproline content and Sirius-red-staining (Trebicka et al., 2010). In conclusion, these markers reflect the release of ECM-fragments into circulation and probably mirror dynamics of fibrogenesis. Thus, they are related to disease activity and may potentially also carry prognostic information. Therefore, they should be tested for monitoring the effect of antifibrotic treatment in human diseases.

### Conflict of interest statement

Diana J. Leeming, Morten A. Karsdal, and Mette J. Nielsen are employees of Nordic Bioscience, a company engaged in the development of biochemical markers. Morten A. Karsdal is stockholder of Nordic Bioscience. The other authors declare that the research was conducted in the absence of any commercial or financial relationships that could be construed as a potential conflict of interest.
